# 3D-Printed Coaxial Hydrogel Patches with Mussel-Inspired Elements for Prolonged Release of Gemcitabine

**DOI:** 10.3390/polym13244367

**Published:** 2021-12-13

**Authors:** Sepehr Talebian, In Kyong Shim, Javad Foroughi, Gorka Orive, Kara L. Vine, Song Cheol Kim, Gordon G. Wallace

**Affiliations:** 1 Intelligent Polymer Research Institute, ARC Centre of Excellence for Electromaterials Science, AIIM Facility, University of Wollongong, Wollongong, NSW 2522, Australia; gwallace@uow.edu.au; 2 Illawarra Health and Medical Research Institute, Wollongong, NSW 2522, Australia; kara@uow.edu.au; 3 Asan Institute for Life Science, Asan Medical Center, University of Ulsan College of Medicine, Seoul 05505, Korea; shimiink@gmail.com; 4 Faculty of Engineering and Information Sciences, University of Wollongong, Wollongong, NSW 2522, Australia; foroughi@uow.edu.au; 5 West-German Heart and Vascular Center, University of Duisburg-Essen, 45122 Essen, Germany; 6 NanoBioCel Research Group, School of Pharmacy, University of the Basque Country (UPV/EHU), 01006 Vitoria-Gasteiz, Spain; gorka.orive@ehu.es; 7 Biomedical Research Networking Centre in Bioengineering, Biomaterials and Nanomedicine (CIBER-BBN), 01006 Vitoria-Gasteiz, Spain; 8 Bioaraba, NanoBioCel Research Group, 01006 Vitoria-Gasteiz, Spain; 9 School of Chemistry and Molecular Bioscience, Molecular Horizons, Faculty of Science, Medicine and Health, University of Wollongong, Wollongong, NSW 2522, Australia; 10 Division of Hepatobiliary and Pancreatic Surgery, Department of Surgery, Asan Medical Center, University of Ulsan College of Medicine, 388-1 Pungnap-2 Dong, Songpa-gu, Seoul 05505, Korea

**Keywords:** hydrogel, 3D printing, drug delivery, cancer

## Abstract

With the aim of fabricating drug-loaded implantable patches, a 3D printing technique was employed to produce novel coaxial hydrogel patches. The core-section of these patches contained a dopamine-modified methacrylated alginate hydrogel loaded with a chemotherapeutic drug (Gemcitabine), while their shell section was solely comprised of a methacrylated alginate hydrogel. Subsequently, these patches were further modified with CaCO_3_ cross linker and a polylactic acid (PLA) coating to facilitate prolonged release of the drug. Consequently, the results showed that addition of CaCO_3_ to the formula enhanced the mechanical properties of the patches and significantly reduced their swelling ratio as compared to that for patches without CaCO_3_. Furthermore, addition of PLA coating to CaCO_3_-containing patches has further reduced their swelling ratio, which then significantly slowed down the release of Gemcitabine, to a point where 4-layered patches could release the drug over a period of 7 days in vitro. Remarkably, it was shown that 3-layered and 4-layered Gemcitabine loaded patches were successful in inhibiting pancreatic cancer cell growth for a period of 14 days when tested in vitro. Lastly, in vivo experiments showed that gemcitabine-loaded 4-layered patches were capable of reducing the tumor growth rate and caused no severe toxicity when tested in mice. Altogether, 3D printed hydrogel patches might be used as biocompatible implants for local delivery of drugs to diseased site, to either shrink the tumor or to prevent the tumor recurrence after resection.

## 1. Introduction

The therapeutic approaches for cancer are very much dependent on the type and state of the disease, and a variety of treatments have been exploited in the clinic setting to date [1]. More specifically, such treatments are aimed to address one or more of the following issues associated with cancer: (i) to shrink inoperable solid tumors to make them operable, (ii) to prevent recurrence of a tumor after surgical resection, and (iii) to eliminate circulating cancer cells in the peripheral blood in patients with metastasis [2]. Along these lines, systemic administration of relevant drugs has been used prevalently to resolve some of the above-mentioned issues, and it even yielded some promising results in the clinic [3]. However, systemic administration of drugs is often linked to undesirable consequences such as high-dose requirement, poor bioavailability, and adverse side effects [4]. This started a scientific movement towards discovering various ways where the drugs could be delivered locally to the tumor site, with the aim of rectifying drawbacks of systemic administration [5]. Consequently, biopolymers entered the fray as candid materials to institute implantable drug delivery systems, designed for local delivery of therapeutics to the diseased site [6,7,8]. Thus, various fabrication technologies were employed to facilitate the making of these biopolymeric drug delivery systems, while each imposed their own set of advantages and disadvantages [6]. Among them, 3D printing technologies offer considerable advantages including reproducibility and ability to design geometrically complex shapes [9]. In addition, 3D printing is compatible with a variety of biopolymers ranging from hydrophobic thermoplastic polymers (such as polycaprolactone; PCL, and polylactic acid; PLA) to hydrophilic hydrogels (such as alginate, chitosan, and gelatine) [10,11]. For instance, Yi et al. fabricated 3D printed patches made from a mixture of PLGA (lactide:glycolide = 85:15) and PCL loaded with Fluorouracil (5-FU), which was aimed for growth suppression of pancreatic cancer [12]. Accordingly, it was shown that the specific geometry (latticed, slant, or triangular) and number of layers could directly affect the release profile of 5-FU. Moreover, these patches could release 5-FU over a period of 4 weeks and, when tested in mice with subcutaneous MIA-PaCa-2 pancreatic tumors, they induced significant reduction in relative tumor size compared to that of empty implanted patches. In another instance, 3D printed scaffolds made from PCL were used for local delivery of Doxorubicin (DOX) to inhibit breast cancer tumor growth [13]. Consequently, the results showed that these scaffolds were capable of releasing DOX over a period of 14 days and, when tested in mice with subcutaneous MDA-MB-231 breast cancer tumors, they were capable of inhibiting tumor growth to a higher degree with less systemic toxicity compared to that of mice who received equivalent dosage of the drug systemically [13]. Based on the existing evidence in the literature, most of the 3D printed cancer drug delivery systems are made from hydrophobic thermoplastic polymers, which indicated the infancy of research in the field of 3D printed hydrogels for cancer drug delivery [6,14,15,16,17,18,19].

We have previously investigated the application of coaxial mussel-inspired hydrogel fibers for delivery of two chemotherapeutic drugs (Gemcitabine and Doxorubicin) and the in vitro release studies showed that these coaxial fibers were capable of releasing Doxorubicin over a period of 21 days [20,21]. However, they did not yield a sustained release of Gemcitabine (GEM) as they released the entire loading in a 10 h time span. The quick release of Gemcitabine from those fibers, as compared to Doxorubicin, was associated with its smaller molecular size. Along similar lines, it is suggested that controlling a hydrogel swelling behavior could decrease its pore size distribution, which can mediate a slower drug release rate from the gel [22]. One of the common approaches to control the swelling of hydrogels is to implement crosslink agents that allow physical or chemical crosslinking of the polymeric chains [23]. For instance, alginate-based hydrogels have been shown to undergo ionic crosslinking with calcium carbonate (CaCO_3_), which directly affected their pore size distribution in a concentration-based manner [24]. Another approach to control the swelling of hydrogels is to subject them to a hydrophobic polymeric coating that could spatially confine the hydrogel boundaries [25]. For instance, drug-loaded alginate disks were successfully coated with PCL, which subsequently led to a reduced swelling of the disks and instigated a slower release of the drug [26].

**Figure 1:**
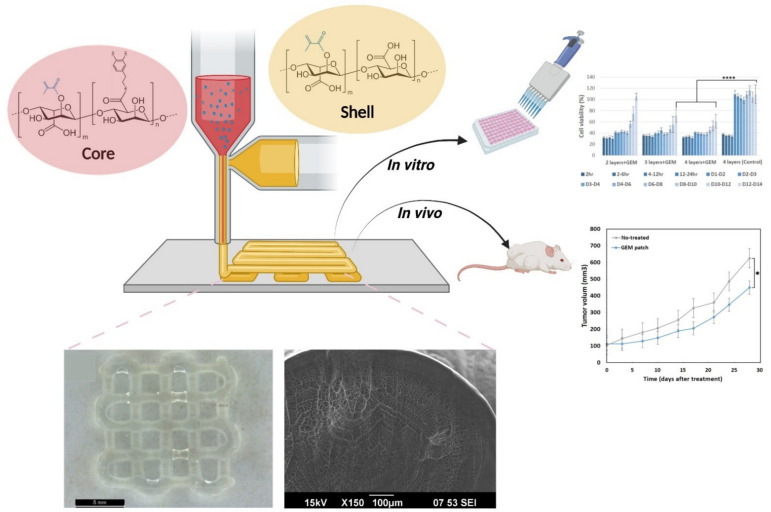
Schematic showing the 3D printing of coaxial hydrogel patches, their morphology, and their subsequent in vitro and in vivo testing. **** *p* = 0.0001, * *p* = 0.05.

Herein, with the purpose of fabricating a suitable platform for prolonged delivery of Gemcitabine (a chemotherapeutic drug), we have 3D printed a novel coaxial hydrogel patch. Particularly, the core-section of these patches contained dopamine-modified alginate methacrylate loaded with Gemcitabine, while their shell section was solely comprised of alginate methacrylate (Figure 1). Subsequently, these patches were further modified with CaCO_3_ cross linker and a PLA coating to facilitate prolonged release of the drug. Accordingly, different properties of the fabricated patches were characterized, including their microstructural morphology (scanning electron microscopy; SEM), tensile properties in both static and dynamic conditions (mechanical tester and dynamic mechanical analysis, respectively), and their swelling behavior. Moreover, the anti-cancer effect of these patches was assessed both in vitro and in vivo by testing them against pancreatic cancer cells. Overall, our proposed approach for achieving a sustained release of Gemcitabine from 3D-printed hydrogel patches has been shown to be effective, and with future development, these patches might become useful as neo-adjuvant or adjuvant therapies in cancer patients.

## 2. Experimental Section

### 2.1. Materials

Alginic acid sodium salt from brown algae (medium viscosity) (CAS No 9005-38-3), *N*-(3-dimethylaminopropyl)-*N*′-ethylcarbodiimide hydrochloride (≥99.0%) (CAS No 25952-53-8), N-hydroxysuccinimide (CAS No 6066-82-6), dopamine hydrochloride (98 %) (CAS No 62-31-7), MES hydrate (≥99.5%) (CAS No 1266615-59-1), methacrylic anhydride (CAS No 760-93-0), calcium carbonate (CaCO_3_; <50 µm size) (CAS No 471-34-1), d-(+)-gluconic acid δ-lactone (GDL) (CAS No 90-80-2), and IRAGACURE D-2959 (CAS No 106797-53-9) were purchased from Sigma Aldrich (Australia). Sodium hydroxide (NaOH) (CAS No 1310-73-2), ethanol (96%) (CAS No 64-17-5), and chloroform (CAS No 67-66-3) were purchased from Chem-Supply (Australia). Poly(DL-lactide) (PURASORB PDL 20) (CAS No 26680-10-4) was purchased from Corbion (Australia). Gemcitabine hydrochloride (CAS No 122111-03-9) was purchased from Focus Bioscience (Australia). Simulated body fluid (SBF) was prepared as explained before [27]. D2O (100%—CIL) (CAS No 7789-20-0) was supplied by Novachem (Heidelberg, West VIC, Australia).

### 2.2. Synthesis and Characterization of Alginate-Methacrylate

Alginate-methacrylate was synthesized using methacrylic anhydride as described previously [28,29]. Briefly, 3 g of alginic acid was dissolved in 300 mL of distilled water (1% *w*/*v*) to which 24 mL of methacrylic anhydride (8% *v*/*v*) was added and the pH was maintained at 8 for 6 h using 5.0 M NaOH solution. Afterwards, the solution was dialyzed (spectra/por membrane tubing; MWCO 12–14 kD) for 7 days and precipitated in ethanol followed by freeze-drying. Successful attachment of methacrylate groups onto the alginate backbone was further confirmed using FTIR (Shimadzu IRPrestige-21 infrared spectrometer) and HNMR (Bruker 400 MHz) spectroscopy.

### 2.3. Synthesis and Characterization of Alginate-Methacrylate-Dopamine

Alginate-methacrylate-dopamine was synthesized using carbodiimide chemistry [30]. Briefly, 1 g of alginate-methacrylate (5 mmol in terms of repeating unit) was dissolved in 100 mL of 0.1 M MES buffer with pH of 5.6. EDC and NHS were separately dissolved in 2 mL of MES buffer (2.5 mmol) and subsequently added to the alginate methacrylate solution. The reaction was allowed to continue for 30 min. Dopamine hydrochloride (at equal concentration to EDC and NHS) was separately dissolved in 2 mL of MES buffer and subsequently added to the mixture. The reaction was allowed to happen for 1 h under nitrogen flushing. Afterwards, the solution was dialyzed (spectra/por membrane tubing; MWCO 12–14 kD) for 7 days against acidic water (pH of 6) and subsequently precipitated in ethanol. The precipitated polymers were next lyophilized using a freeze-dryer. Attachment of dopamine onto alginate-methacrylate backbone was further confirmed using FTIR (Shimadzu IRPrestige-21 infrared spectrometer) and HNMR (Bruker 400 MHz) spectroscopy.

### 2.4. Rheology

All rheology experiments were conducted on a Physica MCR 301 Rheometer (Anton Paar) in parallel plate geometry (50 mm disk, 0.097 mm measuring distance) and at room temperature (23 °C). Flow experiment was performed to evaluate the viscosity of polymer solution (shear rate varying from 1 to 100 s^−1^). Oscillatory experiments as a function of time (at constant shear strain of 1% and constant frequency of 1 Hz) were performed to measure the storage and loss modulus of the hydrogels upon UV irradiation (365 nm, DYMAX BlueWave 75).

### 2.5. 3D Printing of Coaxial Hydrogel Structures

In this study, 2 different formulations of core–shell structures were made: (i) without CaCO_3_ (−CaCO_3_), and (ii) with CaCO_3_ (+CaCO_3_). Two different solutions were used to prepare the core–shell structures. Accordingly, the core aqueous solution contained 6% (*w*/*v*) of alginate-methacrylate-dopamine and 0.05% (*w*/*v*) IRAGACURE D-2959. On the other hand, the shell aqueous solution contained 6% (*w*/*v*) of alginate-methacrylate and 0.05% (*w*/*v*) IRAGACURE D-2959. Of note, for structures with CaCO_3_, 144 mM of CaCO_3_, and 36 mM of GDL were added to both core and shell solutions, based on previously reported values [24]. Additionally, for GEM-containing patches Gemcitabine hydrochloride (50 mM) was added to the core solution. Structures in different geometries (Figure S1, Supplementary Materials) were printed via a GeSiM BioScaffolder (Model 3.1) using a coxial nozzle with a core diameter of 400 µm and a shell diameter of 800 µm. The print parameters as input to the proprietary GeSiM software are listed in Table 1. A UV light (365 nm, DYMAX BlueWave 75) was used to facilitate photo-crosslinking of the structures for both patches and strands.

### 2.6. PLA Coating of 3D Printed Patches

The previously 3D printed patches containing CaCO_3_ were allowed to dry overnight in room temperature, and subsequently dip coated into a 15% (*w*/*v*) solution of Poly(DL-lactide) in chloroform followed by overnight drying at room temperature. The dried coated patches were used for further processing.

### 2.7. Characterization of Printed Structures

The morphology of 3D printed patches was investigated using Leica M205A stereomicroscope. Scanning electron microscopy (SEM) observations were performed using a JEOL JSM-6490LV microscope. SEM images were taken in high vacuum mode at 15 kV operating voltage and a spot size setting of 60. For SEM imaging, the patches were first allowed to completely swell in SBF, then they were cut in half using a razor blade to expose their cross-section and subsequently inserted into special sample holders. The sample holder containing the mounted structure was then immersed into liquid nitrogen for about 45 s. The sample holder was then quickly transferred to the LVSEM for examination. The static tensile properties of strands were assessed using a mechanical tester (EZ-L tester from Shimadzu) at 10 mm·min^−1^ via 50 N and 10 N load cells for dry and wet strands, respectively. The dynamic mechanical properties of strands were evaluated using a dynamic mechanical analysis (DMA 242 E Artemis, NETZSCH). Accordingly, by using a tension clamp, the samples were exposed to a constant strain (0.1%) of increasing frequencies in the range of 0.1 to 10 Hz for 30 min; subsequent storage and loss modulus were recorded and Tan delta (damping factor) values were measured as a ratio of loss modulus to storage modulus. The static compressive properties of the patches in wet state were assessed using a mechanical tester (EZ-L tester from Shimadzu) at 10 mm·min^−1^ via a 50 N load cell. Lastly, the swelling properties of the hydrogel patches were determined by examining their water uptake capacity. The hydrogel patches were incubated in simulated body fluid (SBF) at 37 °C and allowed to fully swell. The swelling ratio was calculated using the following equation: (Ws − Wd)/Wd, where Ws represents the weight of the swollen hydrogel patches and Wd represents the weight of the dried hydrogel patches at the beginning.

### 2.8. Release Studies from Drug Loaded Patches

To measure the Gemcitabine release from the patches, each patch was immersed in 5 mL of SBF solution. The supernatant was collected at certain time points and replaced with fresh SBF. For quantifying the Gemcitabine release, a high-performance liquid chromatography (HPLC, Agilent 1260 infinity) was used. Briefly, samples were filtered through a 0.2 μm syringe membrane filter unit before being injected (10 μL) onto a ZORBAX Eclipse Plus column (4.6 × 100 mm^2^, 5 μm particle size). Using an isocratic elution with a water/acetonitrile (95/5), draw and eject speed of 200 μL/min, pressure 300 bar, Gemcitabine was detected with the UV-Visible detector at 272 nm. The absorption values were converted to concentrations using a previously observed calibration curve. The release results were plotted as mean value of three repeated tests.

### 2.9. In Vitro Cell Studies

All cell lines were authenticated using short tandem repeat (STR) profiling at the Garvan Institute of Medical Research. Cells (MIA-PaCa-2 or PANC-1 cells obtained from ATCC) were cultured in DMEM-High glucose media containing 10% fetal calf serum (FCS) at 37 °C, 95% humidity, and 5% CO_2_ in a Heracell incubator (Kendro Laboratory Products, Alzenau, Germany). When 80% confluence was reached, cells were detached by incubation with 5 mM trypsin/EDTA and harvested after centrifugation in a Heraeus Megafuge 1.0 (Thermo Scientific, Waltham, MA, USA) at 1200 rpm for 5 min at RT. Cells were resuspended in media, and viable cells counted using a hemocytometer and trypan blue staining. Cells were confirmed free of mycoplasma contamination. MIA-Paca-2 and Panc-1 cells were seeded 4 × 10^4^ cells/well in 24-well flat-bottomed plates in complete media (1 mL) containing 1% penicillin/streptomycin and kept in the incubator for 24 h prior to addition of empty or drug loaded patches. After 72 h, the patches were removed from the wells and 40 µL of MTS agent was subsequently added and allowed to react with the cells for 3 h. The absorption of each solution was measured at 490 nm on a microplate reader (SPECTRA max, PLUS). For live cell staining, at 72 h time point the media was removed from the wells and the cells were washed with PBS. Next 500 µL of PBS solution (Containing 2.5 µL of Calcein AM and 1 µL of Propidium Iodide; Sigma Aldrich) was added to the wells and the plates were incubated for 15 min, after which the staining mixture was removed from the wells and replaced with fresh PBS. Immediately after, the IncuCyte ZOOM system (Essen BioScience, Ann Arbor, MI, USA) at 10× magnification, with green (live cells) and red (dead cells) filters, was used to image the cells.

To confirm the long-term therapeutic effect on inhibition of cancer cell growth, the effect of the medium supernatant of the drug loaded or empty patches on the viability of the human pancreatic cancer cell line, MIA-PaCa-2 cell line, was assessed using a cell counting kit-8 method (Sigma, St. Louis, MO, USA) based on manufacturer’s guideline. MIA-PaCa-2 cells were cultured in RPMI-1640 medium (Gibco) supplemented with 10% *v/v* fetal bovine serum (Gibco, Waltham, MA, USA), 100 U/mL penicillin, and 100 μg/mL streptomycin (Invitrogen, Carlsbad, CA, USA) at 37 °C in 5% CO_2_ in a humidified incubator. Cells were plated at a density of 3 × 10^3^ cells per well in a 96-well culture plate and incubated for 24 h before drug treatment. To assess the therapeutic effect of the drug released from the patches, the patches were incubated in complete culture media at 37 °C in 5% CO_2_ in a humidified incubator and then the supernatant was collected at determined intervals. At day 1 after cell seeding, we changed the media to corresponding supernatants. Cell viability at each time point was determined using a cell counting kit-8 assay (CCK-8; Sigma Aldrich, Burlington, MA, USA) according to the manufacturer’s guideline.

### 2.10. In Vivo Studies

To confirm the in vivo effects of the Gemcitabine-loaded patches (PLA coated patches containing CaCO_3_), a subcutaneous tumor model was established using MIA PaCa-2 cells as previously described [20]. MIA PaCa-2 cells were harvested (2 × 10^6^) and resuspended in PBS mixed with Matrigel (1:1 ratio). To prepare the xenograft, tumors were developed in 6-weekold male nude mice by injecting MIA PaCa-2 cells subcutaneously into the right posterior flank of mice. All mouse experiments were within the guidelines of the protocol and were reviewed by the Institutional Animal Care and Use Committee of Asan Institute for Life Science. Tumor growth was recorded twice a week in three dimensions using a digital caliper. Tumor volume was calculated as ((length × width × height)/2) and reported in mm^3^. Tumors were grown for 11–12 days until average tumor volume 100 mm^3^. Mice were randomly divided into 4 groups at day 11 or 12 after subcutaneous cancer-cell inoculation; group I: implanted with drug free 2-layered patches (PLA coated patches containing CaCO_3_); group II: implanted with gemcitabine loaded 2-layered patches (PLA coated patches containing CaCO_3_); group III: implanted with drug free 4-layered patches (PLA coated patches containing CaCO_3_); and group IV: implanted with gemcitabine loaded 4-layered patches (PLA coated patches containing CaCO_3_). All experimental groups started with *n* = 6 mice at the time of treatment initiation. To insert the patches in the mouse, the patches were sterilized in 70% alcohol solution and then wash in the PBS solution twice. Wetted patches were directly attached under the solid tumor. Tumor growth was observed over a period of 4 weeks. The therapeutic effect and anticancer activity of local drug delivery were determined by tumor volume change.

### 2.11. In Vivo Toxicity

Retro-orbital blood collection was performed for hematology determinations in tubes with anticoagulants (EDTA-2 K) on day 3. Hematology determinations included white-blood cell (WBC) count and differential leucocyte count (neutrophils, lymphocytes, and monocytes) using an Advia 120 Hematology Analyzer (Bayer Healthcare, Myerstown, PA, USA).

### 2.12. Statistical Analysis

Statistical significance was determined using a two-way ANOVA with a Sidak post-test or paired multiple *t*-test (GraphPad Prism V 6.0; San Diego, CA, USA). *p* values < 0.05 were considered statistically significant. * *p* = 0.05, ** *p* ≤ 0.01, *** *p* = 0.001, and **** *p* = 0.0001. Values are reported as the average ± standard deviation.

## 3. Results and Discussion

### 3.1. Chemical Characterization of the Synthesized Polymers

**Figure 2:**
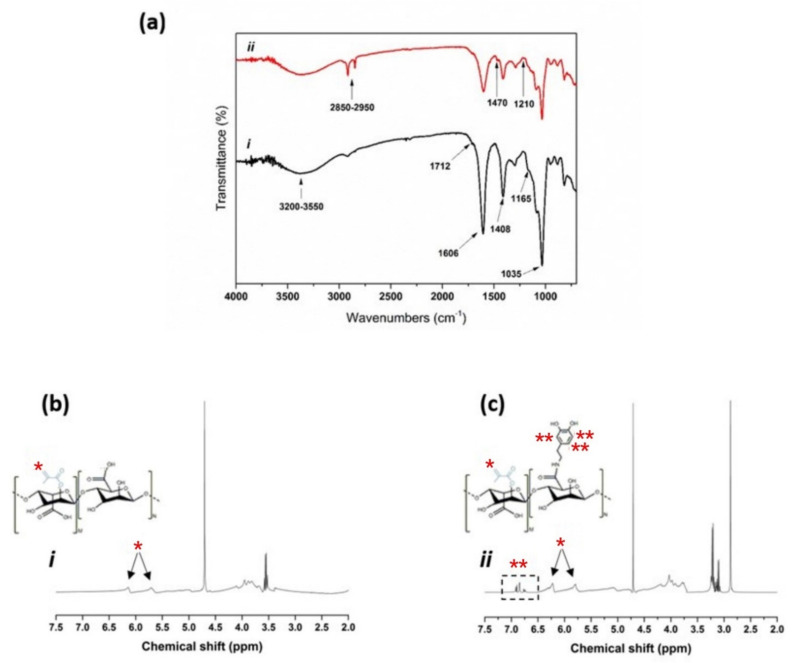
Chemical characterization of as–synthesized polymers including, (**a**) FTIR and (**b**,**c**) HNMR spectra of (i) alginate–methacrylate, and (ii) alginate–methacrylate–dopamine. * indicating the methacrylic protons, ** indicating the dopamine protons.

Initially, the chemical structure of the synthesized polymers was assessed using Fourier Transform Infrared (FTIR) and Proton nuclear magnetic resonance (HNMR) spectroscopies and results are shown in Figure 2. Accordingly, the FTIR spectra of both alginate-methacrylate and alginate-methacrylate-dopamine (Figure 2a) showed the characteristic peaks of alginate at 1035 cm^−1^, 1408 cm^−1^, 1606 cm^−1^, and 3350 cm^−1^ associated with C-O stretching vibration, COO-symmetric and -asymmetric stretching vibration, and –OH stretching vibrations, respectively [31]. More specifically, both spectra also showed two more peaks at 1165 cm^−1^ and 1712 cm^−1^ attributed to, respectively, C=O and C-O groups of the esters resulting from the grafting of the methacrylate units [32]. Additionally, the alginate-methacrylate-dopamine spectrum showed the appearance of three new peaks (due to attachment of dopamine) at 1210 cm^−1^, 1470 cm^−1^, and 2850–2950 cm^−1^ assigned to C–N stretching vibrations, N–H deformation, and C-H stretching vibrations, respectively [33,34]. Additionally, HNMR analysis of alginate-methacrylate (Figure 2b) proved the grafting of methacrylate groups by appearance of double peaks (vinyl) in the double bond region (5.5–6.5 ppm) [32]. Furthermore, the relative integration of methyl protons (of methacrylate groups) to anomeric protons of the glucose ring of alginate (4.9 ppm) were used to determine the degree of substitution which was 42% (in terms of alginate repeating units).

In a similar manner, HNMR analysis of alginate-methacrylate-dopamine (Figure 2c) showed the mentioned double peaks in the double bond region (5.5–6.5 ppm) associated with methacrylate groups, along with appearance of catechol protons at around 7 ppm associated with the dopamine [35]. Further, the relative integration of catechol protons (6.6–7 ppm) to anomeric protons of the glucose ring of alginate (4.9 ppm) were used to determine the degree of substitution which was measured to be 18% (in terms of alginate repeating units). Overall, these results suggested that alginate-methacrylate and alginate-methacrylate-dopamine were successfully synthesized.

### 3.2. Rheometry

**Figure 3:**
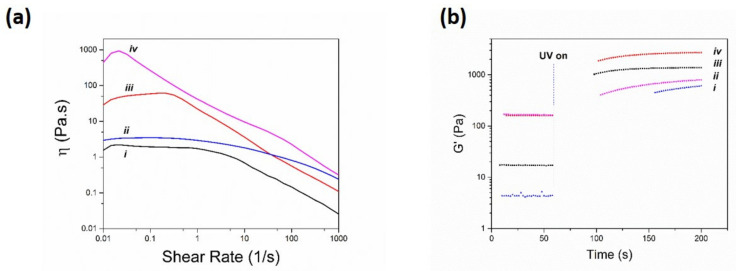
Rheometry of printing solutions including (**a**) Viscometry of 6% (*w*/*v*) solution of (i) alginate–methacrylate, (ii) alginate–methacrylate–dopamine, (iii) alginate–methacrylate + CaCO_3_, and (iv) alginate–methacrylate–dopamine + CaCO_3_. (**b**) Oscillatory rheology of 6% (*w*/*v*) solutions (containing 0.05% (*w*/*v*) IRAGACURE D-2959) of (i) alginate–methacrylate–dopamine, (ii) alginate–methacrylate–dopamine + CaCO_3_, (iii) alginate–methacrylate, and (iv) alginate–methacrylate + CaCO_3_.

Considering the essential role of polymer rheology in subsequent properties of the 3D printed structure [36], viscoelastic properties of all printing solutions were assessed using a rheometer and the results are shown in Figure 3.

Accordingly, both alginate-methacrylate (Figure 3a(i)) and alginate-methacrylate-dopamine (Figure 3a(ii)) solutions demonstrated shear thinning behavior as their viscosities gradually decreased with an increase in the shear rate, yet alginate-methacrylate-dopamine solution showed a higher viscosity throughout the measurement range which was speculated to be a result of noncovalent interactions of dopamine moieties among each other [37]. Most remarkably, after addition of CaCO_3_ the viscosity of both alginate-methacrylate (Figure 3a(iii)) and alginate-methacrylate-dopamine (Figure 3a(iv)) solutions increased significantly due to ionic interaction between Ca^2+^ ions and alginate backbone, however alginate-methacrylate-dopamine solution showed a higher level of increase in its viscosity which was assumed to be due to catechol-ion interactions through formation of a bis-complex [38]. Moreover, both alginate-methacrylate and alginate-methacrylate-dopamine solutions containing CaCO_3_ (Figure 3a(iii,iv)), showed a more pronounced shear-thinning behavior when compared to their counterparts without CaCO_3_ (Figure 3a(i,ii)). This is due to disruption of calcium-alginate egg-box model and calcium-dopamine bis-complex upon increasing the shear-rate [39]. To further evaluate the UV crosslinking-ability of the 3D printing solutions, oscillatory experiments (as a function of time) upon UV irradiation were conducted, and the results are shown in Figure 3b. Accordingly, both alginate-methacryate (Figure 3b(iii)) and alginate-methacrylate-dopamine (Figure 3b(i)) solutions experienced a significant jump in their storage modulus upon initiating the UV irradiation, an indication of UV cross-linking of both solutions. Of note, alginate-methacrylate-dopamine experienced a slightly lower storage modulus after UV irradiation when compared to that of alginate-methacrylate, which could be attributed to the effect of dopamine moieties in the polymer absorbing part of the UV rays [40]. Additionally, the addition of CaCO_3_ to alginate-methacrylate (Figure 3b(iv)) and alginate-methacrylate-dopamine (Figure 3b(ii)) solutions caused a slight increase in their corresponding storage modulus associated with presence of ionic bonds in the formulations. Overall, it was shown that all solutions had shear-thinning behaviors and were UV-crosslinkable, making them suitable for extrusion printing.

### 3.3. Morphological Analysis of 3D Printed Patches

**Figure 4:**
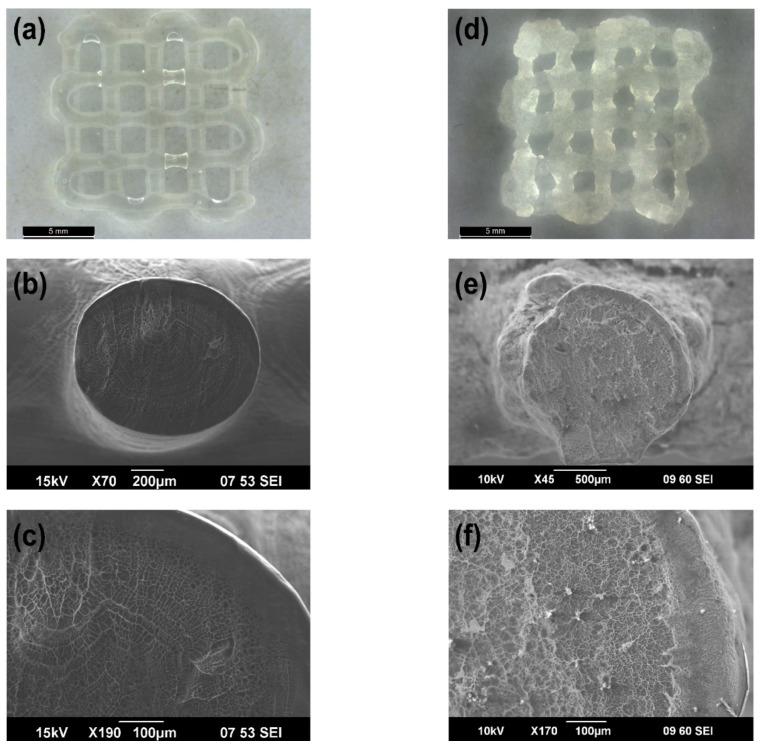
(**a**) Light microscopy and (**b**,**c**) SEM images of 3D printed coaxial patches without CaCO_3_. (**d**) Light microscopy and (**e**,**f**) SEM images of 3D printed coaxial patches with CaCO_3_.

With the aim of studying the morphology of the 3D printed patches, light microscopy and scanning electron microscopy were implemented and the results are shown in Figure 4 and Figure S2 (Supplementary Materials). First, we have evaluated the morphology of 3D printed structure in the absence of CaCO_3_ cross linker (Figure 4a,c). Accordingly, it was shown that these patches possessed intricate structures with well-ordered dimensions, and their cross-section SEM images revealed their perfectly circular coaxial microstructure where the shell thickness was in the range of 70–84 µm. Of interest, the shell showed a less porous structure as compared to that of the core, probably due to absorption of a portion of UV light by the dopamine moiety in the core which hindered their further chemical cross-linking (as it was observed in rheology studies). Next, we have examined the morphology of 3D printed patches that contained CaCO_3_ cross linker (Figure 4d,f). The results showed that these patches contained opaque structures with rough edges due to high viscosity of their precursor solutions and their cross-section SEM images further revealed their almost circular coaxial microstructure with occasional CaCO_3_ agglomerates, where the shell thickness was in range of 120–145 µm. Lastly, the morphology of PLA coated 3D printed patches containing CaCO_3_ cross linker was examined, and the results are shown in Figure S2 (Supplementary Materials). Correspondingly, the PLA coating was hard to detect from the light microscopy image, however their cross-section SEM images clearly showed the PLA coating around a single strand of the structure with a thickness in range of 50–90 µm. Of note, the coaxial structure was hard to observe in these structures as the PLA coating avoided full swelling of the hydrogel components. Additionally, the partial deformation of the encapsulated 3D printed structure in this SEM image was a result of cutting procedure to expose their cross-section for imaging purposes. Overall, the results showed that addition of CaCO_3_ cross linker to the patches caused changes to the microstructure of the patches due to interactions between Ca^2+^ and alginate backbone. Additionally, it was shown that PLA coating did not have any negative effect on the microstructure of the patches.

### 3.4. Mechanical Properties of the 3D Printed Patches

To further assess the effect of CaCO_3_ cross linker addition on the subsequent mechanical properties of the fabricated structures, we have specifically printed single coaxial strands (as described in Experimental section and shown in Figure S1a, Supplementary Materials) and applied them to either static or dynamic tensile stress. Thus, measurements in static state were carried out for strands in both dry and wet state and the results are shown in Figure S3, Supplementary Materials. Correspondingly, in dry state, no significant difference was observed in tensile strength values of the strands without CaCO_3_ (30.3 ± 3.3 MPa) and the ones with CaCO_3_ (29.3 ± 2.4 MPa). However, strands with CaCO_3_ had higher modulus values (1.1 ± 0.1 GPa) when compared to those of strands without CaCO_3_ (0.7 ± 0.05 GPa). In wet state a significant difference was observed in both tensile strength and modulus values of the strands with CaCO_3_ (0.096 ± 0.002 MPa and 0.17 ± 0.02 MPa, respectively) as compared to those of strands without CaCO_3_ (0.05 ± 0.005 MPa and 0.025 ± 0.007 MPa, respectively). The obtained results suggested that the addition of CaCO_3_ cross linker has led to improvements in mechanical properties of the strands especially in wet state, and the corresponding values were comparable to that of other printed hydrogel strands in the literature [41,42].

**Figure 5:**
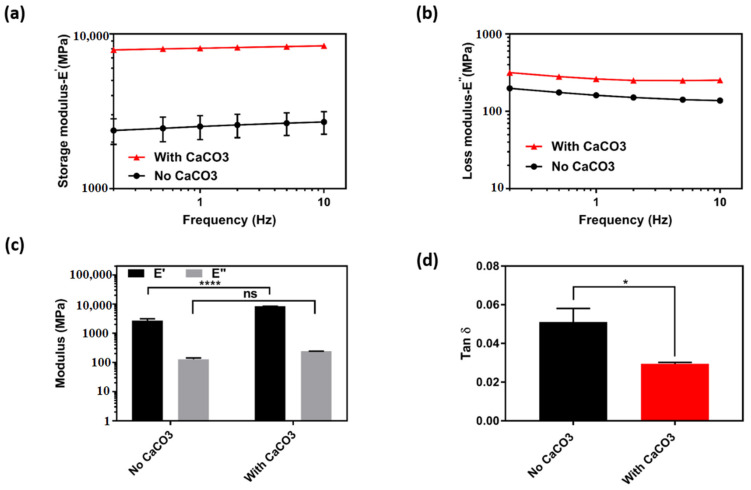
Dynamic mechanical analysis of 3D printed coaxial strands by using a tension clamp. The samples were exposed to a constant strain (0.1%) of increasing frequencies in the range of 0.1 to 10 Hz for 30 min. (**a**) Storage modulus and (**b**) loss modulus of strands at different frequencies. (**c**) Storage and loss modulus at the frequency of 10 Hz. (**d**) Tan delta (damping factor) at the frequency of 10 Hz (*n* = 3, mean ± SD) (* *p* = 0.05, **** *p* = 0.0001).

To gain a better insight into dynamic mechanical properties of the strands, an oscillating tension test (at a constant strain of 0.1%) in a frequency range of 0.1–10 Hz was conducted (Figure 5). The obtained results showed that for both formulations (with and without CaCO_3_) storage modulus values were significantly higher than loss modulus values over the entire frequency range, indicating the prominence of elastic behavior over viscous behavior in all the formulations (Figure 5c). Moreover, as shown in Figure 5a, strands with CaCO_3_ showed a significantly higher storage modulus (8159.5 ± 182.7 MPa) over the entire frequency range when compared to that of strands without CaCO_3_ (2233.0 ± 119.3 MPa). As a result, strands with CaCO_3_ showed to have a significantly lower damping factor (0.0295 ± 0.0007) in comparison to that of strands without CaCO_3_ (0.051 ± 0.007) (Figure 5d). Overall, the measurements in dynamic conditions showed that strands with CaCO_3_ had a more elastic response to oscillating stress when compared to strands without CaCO_3_, which was further attributed to calcium–alginate and calcium–dopamine interactions that were previously observed in rheology measurements. Lastly, given the superior tensile properties of structures with CaCO_3_, a static compression test was applied to 2-, 3-, and 4-layered 3D printed patches that were made from the same formulation and the results are shown in Figure S4, Supplementary Materials. Consequently, a common trend was observed where the strength of the patches increased in accordance with their number of layers, as the 4-layered structures showed to withstand the most amount of compression force amongst others 10,000.

### 3.5. Gemcitabine Release Studies

Given the role of polymer’s swelling on their subsequent release of drugs, the swelling ratio values of the tested patches were measured, and the results are shown in Figure S5a. Accordingly, the addition of CaCO_3_ has significantly reduced the swelling ratio of the patches from 42.9 ± 1.0 to 17.9 ± 0.6, which was attributed to calcium–alginate and calcium–dopamine interactions. Moreover, the addition of a PLA coating to CaCO_3_-contaning patches further reduced their swelling ration down to 1.2 ± 0.5, which was due to hydrophobic nature of the PLA coating, which hindered fast penetration of water into the structures and subsequently prevented them from reaching their maximum swelling.

**Figure 6:**
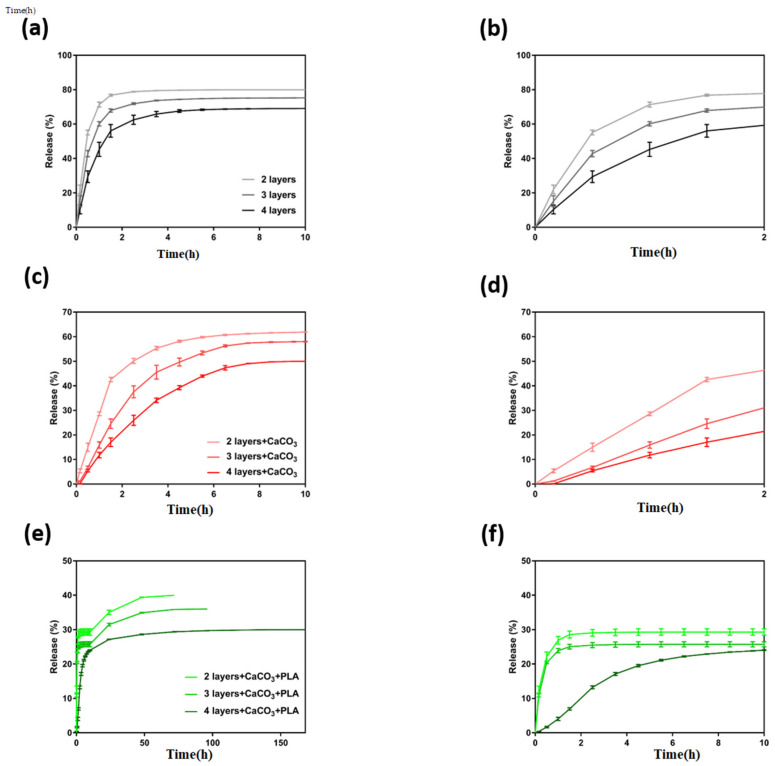
In vitro Gemcitabine release studies from various 3D printed coaxial patches including (**a**,**b**) patches without CaCO_3_, (**c**,**d**) patches with CaCO_3_, and (**e**,**f**) PLA coated patches with CaCO_3_. (*n* = 3, mean ± SD).

Next, in vitro release of Gemcitabine from three different formulations of 3D printed patches (without CaCO_3_, with CaCO_3_, and PLA coated with CaCO_3_) were assessed as described in experimental section and the results are shown in Figure 6.

Accordingly, patches without CaCO_3_ (Figure 6a,b) showed a burst release of the drug in the first 2 h of the experiment where 78.8 ± 0.2%, 71.9 ± 0.5%, and 62.5 ± 2.6% of the drug was released from 2-, 3-, and 4-layered structures, respectively. However, all these patches reached a plateau level at the 4 h of the release and they failed to yield a sustained release of the drug. Subsequently, patches with CaCO_3_ (Figure 6c,d) were tested and they collectively showed a slower release profiles compared to obtained results from the patches without CaCO_3_. Patches with CaCO_3_ still showed a burst release of the drug in the first 2 h of the experiment where 50.1 ± 1.0%, 37.5 ± 2.4%, and 25.9 ± 2.0% of the drug was released from 2-, 3-, and 4-layered structures, respectively. However, these patches also failed to provide a sustained release of Gemcitabine, as their release profiles reached a plateau level at 6 h of release. Next, PLA coated patches containing CaCO_3_ (Figure 6e,f) were tested, and they collectively showed a slower release profiles compared to obtained results from the patches with only CaCO_3_. Correspondingly, these patches have released Gemcitabine in a peculiar manner over two different stages: (i) initially, Gemcitabine was released with a faster rate in the first 10 h of the experiment where 29.3 ± 0.9%, 25.7 ± 0.7%, and 24.0 ± 0.07 of the drug was released from 2-, 3-, and 4-layered structures, respectively. (ii) This was followed by a slower release of the drug which lasted 3 days (40.0%), 4 days (36.0%), and 7 days (30.0%) for 2-, 3-, and 4-layered structures, respectively. The obtained results suggested that PLA coated patches containing CaCO_3_ had the slowest Gemcitabine release profile, and specifically 4-layered structures were capable of releasing the drug over a period of 7 days, which was longer than that from other hydrogels reported in the literature [27,43,44,45,46,47,48]. Of note, we have measured the amount of Gemcitabine that was released from PLA coated patches containing CaCO_3_ (using the collected HPLC data) after 14 days in vitro, and the collected values for 2-, 3-, and 4-layered structures were equal to 250 ± 10 µg, 350 ± 5 µg, and 450 ± 20 µg, respectively. Overall, based on the obtained results, PLA coated patches containing CaCO_3_ were chosen for further in vitro cell studies.

### 3.6. In Vitro Biocompatibility of the 3D Printed Patches

**Figure 7:**
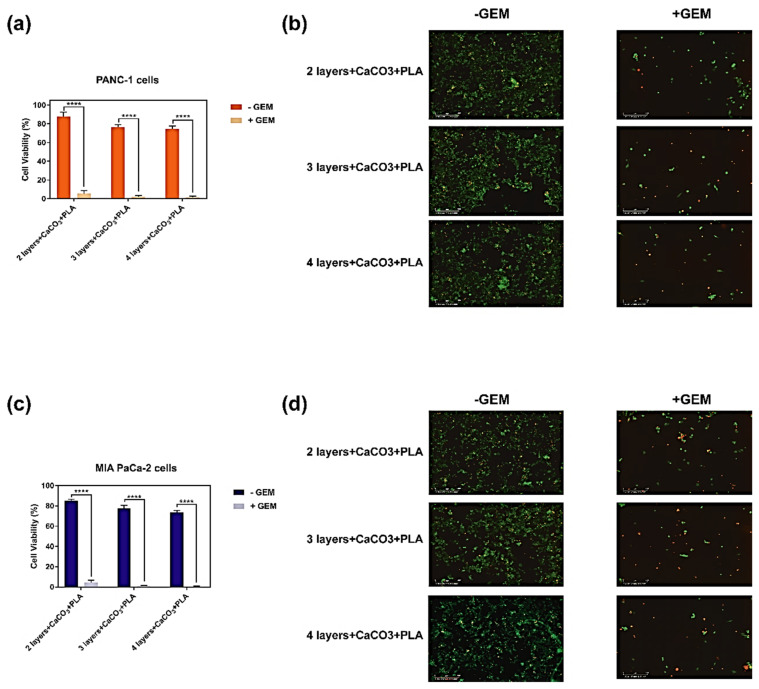
In vitro biocompatibility of PLA coated 3D printed coaxial patches containing CaCO_3_ either without Gemcitabine (− GEM), or loaded with Gemcitabine (+ GEM). (**a**) MTS cell viability assay of PANC-1 human pancreatic adenocarcinoma cells when treated with the patches for 72 h. (**b**) Corresponding live/dead cell staining of treated PANC-1 human pancreatic adenocarcinoma cells. (**c**) MTS cell viability assay of MIA-PaCa-2 human pancreatic adenocarcinoma cells when treated with the patches for 72 h. (**d**) Corresponding live/dead cell staining of treated MIA-PaCa-2 human pancreatic adenocarcinoma cells. Values are the mean (±SEM) of quadruplicate. **** *p* = 0.0001.

With the purpose of evaluating the biocompatibility of the PLA coated patches containing CaCO_3_, they have been tested in vitro on two human pancreatic cancer cell lines (MIA-PaCa-2 and PANC-1) for a period of 72 h and the results are shown in Figure 7. Accordingly, when empty patches, indicated as “—GEM”, were tested against both cell lines they showed > 80% viability (Figure 7a,c), which was further confirmed with the live/dead imaging of the treated cells (Figure 7b,d). However, when Gemcitabine loaded patches, indicated as “+ GEM”, were tested against both cell lines they caused a significantly low viability in the cells (<6%) (Figure 7a,c), which was evident from low density of viable cells in the corresponding live/dead images taken from treated cells (Figure 7b,d). Overall, these results showed that empty patches did not cause any significant toxicity to the cells, but long-term therapeutic effects of these drug loaded patches is essential to evaluate their potential in preventing the cancer cell growth.

### 3.7. In Vitro Therapeutic Effect of the 3D Printed Patches

**Figure 8:**
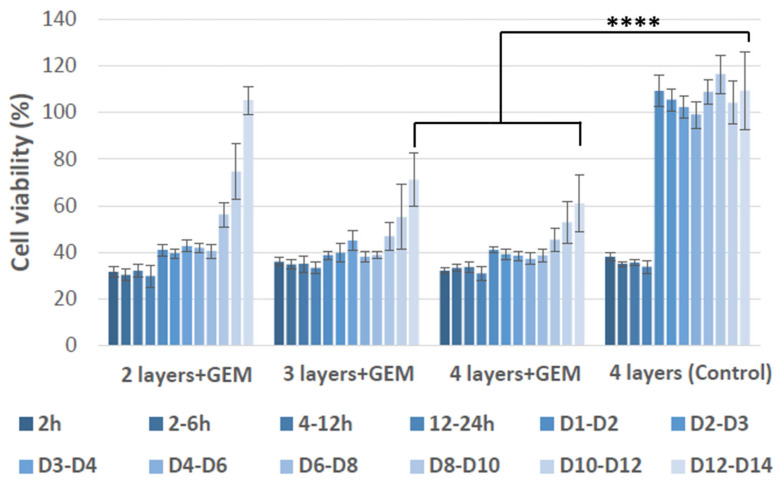
Therapeutic effect of PLA coated 3D printed coaxial patches containing CaCO_3_, with Gemcitabine (+GEM) or without Gemcitabine (control), on inhibition of MIA-PaCa-2 pancreatic cancer cell growth. Values are the mean (±SEM) of quadruplicate. D in the figure legend denotes days of treatment. **** *p* = 0.0001.

Next, the long-term therapeutic effect of Gemcitabine loaded patches (PLA coated patches containing CaCO_3_) were examined in vitro by testing them against MIA-PaCa-2 pancreatic cancer cells and the results are shown in Figure 8.

Four different patches were tested for this purpose, including Gemcitabine loaded 2-, 3-, and 4-layered patches, as well as empty 4-layered patches which were used as a control. Consequently, the results showed that empty 4-layered patches showed some level of toxicity to the cells for the first 24 h of testing (cell viability dropped to almost 40%), which the cells then recovered from in the following days to a point where only viable cells remained at the end of the 14th day. Most significantly, Gemcitabine loaded 2-layered patches were only capable of inhibiting cancer cell growth for 8 days, whereas 3- and 4-layered patches succeeded to prevent the cancer cell growth up to 10 days before the cells started to increase again. More specifically, after 14 days, cells treated with Gemcitabine loaded 2-layered structures fully regained their viability, whereas cells treated with Gemcitabine loaded 3- and 4-layered patches had a viability of 72 ± 10% and 61 ± 9% at day 14th of the experiment, respectively. The obtained data showed that by adjusting the number of layers in the drug loaded patches, one can control the duration of their therapeutic effect. Similar results were obtained when the Gemcitabine loaded patches were tested against Panc-1 pancreatic cancer cell (Figure S6). However, these cells showed more resilience to the Gemcitabine treatment as compared to MIA-PaCa-2 cells, in accordance with previous observations by other groups [49,50]. Overall, the results suggested that 3- and 4-layered patches had better performance in inhibiting pancreatic cancer cell growth when compared to 2-layered structures. This could be associated with higher concentration of drug presented in 3- and 4-layered structures, as well as their slower gemcitabine release profile.

### 3.8. In Vivo Therapeutic Effect of the 3D Printed Patches

**Figure 9:**
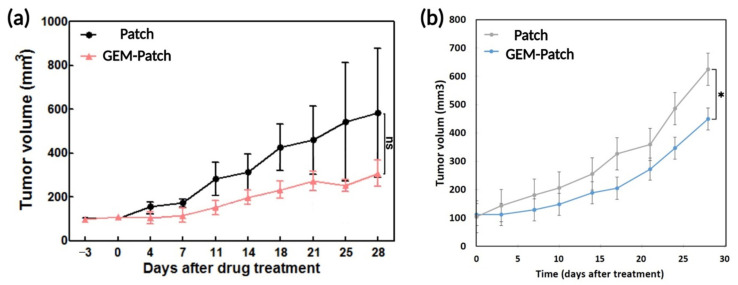
In vivo performance of Gemcitabine-loaded patches (PLA coated patches containing CaCO_3_) in MIA PaCa-2 mice xenografts compared to empty patches (*n* = 6 in each group, * *p* = 0.05). (**a**) Tumor volume in animals treated with empty 2-layered patches (Patch), and Gemcitabine-loaded 2-layered patches (GEM-Patch). (**b**) Tumor volume in animals treated with empty 4-layered patches (Patch) and Gemcitabine-loaded 4-layered patches (GEM-Patch).

Subsequently, in vivo experiments were carried out for a period of 4 weeks using a murine subcutaneous xenograft tumor model using pancreatic cancer cell line MIA PaCa-2 (Figure 9). Following tumor formation, the Gemcitabine loaded patches (PLA coated patches containing CaCO_3_) were implanted under the tumor region, through a small incision in the middle of the back of the mice, and after 4 weeks the animals were sacrificed, and their tumor size were measured. Accordingly, no significant difference in tumor volume was observed when animals were treated with either empty patches or Gemcitabine-loaded 2-layes patches (Figure 9a). This observation was in line with our previously obtained in vitro analysis which showed the inability of 2-layered patches to significantly inhibit the tumor growth. However, Gemcitabine-loaded 4-layered structures could slow down the tumor growth more effectively compared to 2-layered patches (Figure 9b).

Nevertheless, neither of the Gemcitabine-loaded patches were able to completely inhibit the tumor growth. Similar in vivo results were obtained from other Gemcitabine-loaded implants, indicating that sustained and prolonged release of gemcitabine is associated with minimal therapeutic effects [51,52]. Regardless, to assess the in vivo toxicity effect of the patches, various parameters in the blood of the mice were analyzed (Table 2). The obtained results for mice treated with Gemcitabine-loaded patches showed no significant difference from the hematological values (i.e., white blood cell, red blood cell, hemoglobin, platelet, neurophil, and lymphocyte count) of a normal mouse, suggesting that patches did not produce any significant changes in the hematology of mice, implying that patches may be a safe delivery platform for in vivo use.

## 4. Conclusions

In this study, for the first time, 3D printed coaxial hydrogel patches were fabricated for prolonged release of Gemcitabine. Specifically, the core-section of these patches contained alginate-methacrylate-dopamine loaded with Gemcitabine, while their shell section was solely comprised of alginate-methacrylate. Consequently, with the aim of enhancing the Gemcitabine release profile, the patches were first modified with CaCO_3_ cross linker in both core and shell. Subsequently, characterization of these patches showed that addition of CaCO_3_ led to significant improvements in their mechanical properties (in both static and dynamic conditions) and swelling behavior when compared to those of original hydrogel patches. However, patches containing CaCO_3_ failed to yield a prolonged release of Gemcitabine in vitro, as they released the entire drug in the first 10 h of the experiment. As a result, the effect of addition of a PLA coating to these patches on their subsequent drug release profile was studied. Remarkably, the addition of a PLA coating led to a significant reduction in swelling of the hydrogel patches, which in turn translated into a prolonged release of the drug in vitro over a span of 7 days. The PLA coated patches showed good level of biocompatibility in vitro when tested against two pancreatic cancer cell lines (MIA-PaCa-2 and PANC-1). Moreover, in a 14 day in vitro experiment against MIA-PaCa-2 cells, the 3- and 4-layered PLA coated drug loaded patches succeeded in preventing the growth of cancer cells over the entire testing period. Subsequently, in vivo testing of Gemcitabine-loaded patches revealed that 4-layered structures were capable of slowing down the tumor growth rate without any severe side effects in the mice. However, these patches came up short in reducing the tumor size. These observations indicated that sustained and gradual release of Gemcitabine might not yield the best therapeutic efficacy, and probably pulsatile release patterns could lead to more effective treatments. In conclusion, these PLA coated 3D printed patches might be used as biocompatible implants for local delivery of other chemotherapeutics to diseased sites, to either shrink the tumor or to prevent the tumor recurrence after resection.

## Figures and Tables

**Table 1:** Parameters used for fabrication of the printed structures.

Parameter	Patch	Strand
Printing pressure (Core)	50 kPa (−CaCO_3_) or 200 kPa (+CaCO_3_)	50 kPa (−CaCO_3_) or 200 kPa (+CaCO_3_)
Printing pressure (Shell)	30 kPa (−CaCO_3_) or 150 kPa (+CaCO_3_)	30 kPa (−CaCO_3_) or 150 kPa (+CaCO_3_)
Printing speed	3.5 mm/s	3.5 mm/s
Layer height	0.4 mm	0.4 mm
Distance between strands	3 mm	-
Turn between layers	90°	-
Pause between layers	10 s	-

**Table 2:** Hematologic parameters from MIA-PaCa-2 tumor bearing mice in various treatment groups. Data are presented as average ± SD (*n* = 6). WBC: white blood cell, RBC: red blood cell, HGB: hemoglobin, HCT: hematocrit, MCV: mean corpuscular volume, MCH: mean corpuscular hemoglobin, MCHC: mean corpuscular hemoglobin concentration, RDW: red cell distribution width, HDW: hemoglobin distribution width, PLT: platelet, MPV: mean platelet volume, Neut: neutrophils, Lymp: lymphocytes, Mono: monocytes, BASO: basophil, and Luc: large unstained cells.

	Not-Treated	2L GEM Patch (Full Size, 3 Days)	4L GEM Patch (Full Size, 3 Days)
WBC (10e^3^/μL)	5.88 ± 1.29	5.26 ± 0.57	6.77 ± 0.76
RBC (10e^6^/μL)	10.09 ± 0.19	10.98 ± 1.10	9.28 ± 2.62
HGB (g/dL)	15.03 ± 0.12	16.25 ± 1.21	14.75 ± 3.89
HCT (%)	49.23 ± 0.23	51.65 ± 4.6	48.10 ± 8.77
MCV (fL)	48.77 ± 1.01	47.05 ± 0.49	52.65 ± 5.44
MCH (Pg)	14.90 ± 0.30	14.85 ± 0.35	15.90 ± 0.28
MCHC (g/dL)	30.53 ± 0.29	31.50 ± 0.57	30.35 ± 2.62
CHCM (g/dL)	30.07 ± 0.29	30.70 ± 0.71	30.70 ± 1.70
RDW (%)	13.83 ± 0.51	14.30 ± 0.85	20.75 ± 10.39
HDW (%)	2.02 ± 0.08	2.45 ± 0.07	2.51 ± 0.71
PLT (10e^3^/μL)	1114.67 ± 24.58	801.00 ± 149.91	1110.50 ± 109.60
MPV (fL)	8.20 ± 0.2	7.40 ± 1.7	6.50 ± 0.57
%NERU	42.77 ± 8.77	14.05 ± 3.75	38.80 ± 4.38
%LYMPH	49.10 ± 9.85	36.60 ± 1.62	53.70 ± 2.97
%MONO	3.80 ± 1.35	1.75 ± 1.48	1.55 ± 0.49
%EOS	2.70 ± 0.40	4.10 ± 0.63	2.00 ± 0.42
%BASO	0.70 ± 0.52	2.80 ± 0.42	1.00 ± 0.00
%LUC	0.9 ± 0.35	1.55 ± 1.20	3.00 ± 1.27

## Supplementary Materials

The following are available online at https://www.mdpi.com/article/10.3390/polym13244367/s1, Figure S1: Design of 3D printed coaxial structures including (a) strands, (b) patches. Figure S2: Light microscopy and SEM images of PLA coated 3D printed coaxial patches containing CaCO_3_. Figure S3: Mechanical properties of 3D printed coaxial strands following a static tensile test, (a–c) Stress-strain curve, tensile strength, and modulus of strands in dry state (*n* = 3, mean ± SD). (d–f) Stress-strain curve, tensile strength, and modulus of strands in wet state (*n* = 3, mean ± SD). (* *p* = 0.05, ** *p* ≤ 0.01). Figure S4. Static compression testing of 3D printed coaxial patches containing CaCO_3_. Figure S5. Swelling ratio of different 3D printed coaxial patches in simulated body fluid (SBF). (b) Mass of PLA coating on the 3D printed coaxial patches containing CaCO_3_, showing no significant difference between 2-, 3-, or 4-layers structures. Figure S6. Therapeutic effect of PLA coated 3D printed coaxial patches containing CaCO_3_, with Gemcitabine (+GEM) or without Gemcitabine (control), on inhibition of MIA-PaCa-2 pancreatic cancer cell growth. Values are the mean (±SEM) of quadruplicate. D in the figure legend denotes days of treatment. ** *p* ≤ 0.01.
