# Supplementary material for: 3D-Printed Coaxial Hydrogel Patches with Mussel-Inspired Elements for Prolonged Release of Gemcitabine
Source: Polymers (Basel). 2021 Dec 13;13(24):4367. doi: 10.3390/polym13244367 (PMC8708853; doi:10.3390/polym13244367)
Supplement: Supplementary file 1 [file polymers-13-04367-s001.zip › polymers-1438429-supplementary.pdf]

3D-Printed Coaxial Hydrogel Patches with Mussel-Inspired Elements for Prolonged Release of Gemcitabine

Sepehr Talebian [1,2,*,†], In Kyong Shim [3,†], Javad Foroughi [4,5], Gorka Orive [6,7,8], Kara L. Vine [2,9], Song Cheol Kim [10,*] and Gordon G. Wallace [1]

Correspondence should be addressed to Dr. Sepehr Talebian (sepehr.talebian@sydney.edu.au) and Prof. Song Cheol Kim (drksc@amc.seoul.kr).

1 Intelligent Polymer Research Institute, ARC Centre of Excellence for Electromaterials Science, AIIM Facility, University of Wollongong, Wollongong, NSW 2522, Australia; gwallace@uow.edu.au
2 Illawarra Health and Medical Research Institute, Wollongong, NSW 2522, Australia, kara@uow.edu.au
3 Asan Institute for Life Science, University of Ulsan College of Medicine and Asan Medical Center, Seoul 05505, Korea; shimiink@gmail.com
4 Faculty of Engineering and Information Sciences, University of Wollongong, Wollongong, NSW 2522 Australia; foroughi@uow.edu.au
5 West-German Heart and Vascular Center, University of Duisburg-Essen, Essen 45122, Germany
6 NanoBioCel Research Group, School of Pharmacy, University of the Basque Country (UPV/EHU), Vitoria-Gasteiz 01006, Spain; gorka.orive@ehu.es
7 Biomedical Research Networking Centre in Bioengineering, Biomaterials and Nanomedicine (CIBER-BBN).Vitoria-Gasteiz 01006, Spain
8 Bioaraba, NanoBioCel Research Group, Vitoria-Gasteiz 01006, Spain
9 School of Chemistry and Molecular Bioscience, Molecular Horizons, Faculty of Science, Medicine and Health, University of Wollongong, Wollongong, NSW 2522, Australia
10 Division of Hepatobiliary and Pancreatic Surgery, Department of Surgery, Asan Medical Center, University of Ulsan College of Medicine, 388-1 Pungnap-2 Dong, Songpa-gu, Seoul, 05505, Korea
* Correspondence: sepehr.talebian@sydney.edu.au (S.T.); drksc@amc.seoul.kr (S.C.K.)
† The first two authors contributed equally

Supplementary Materials

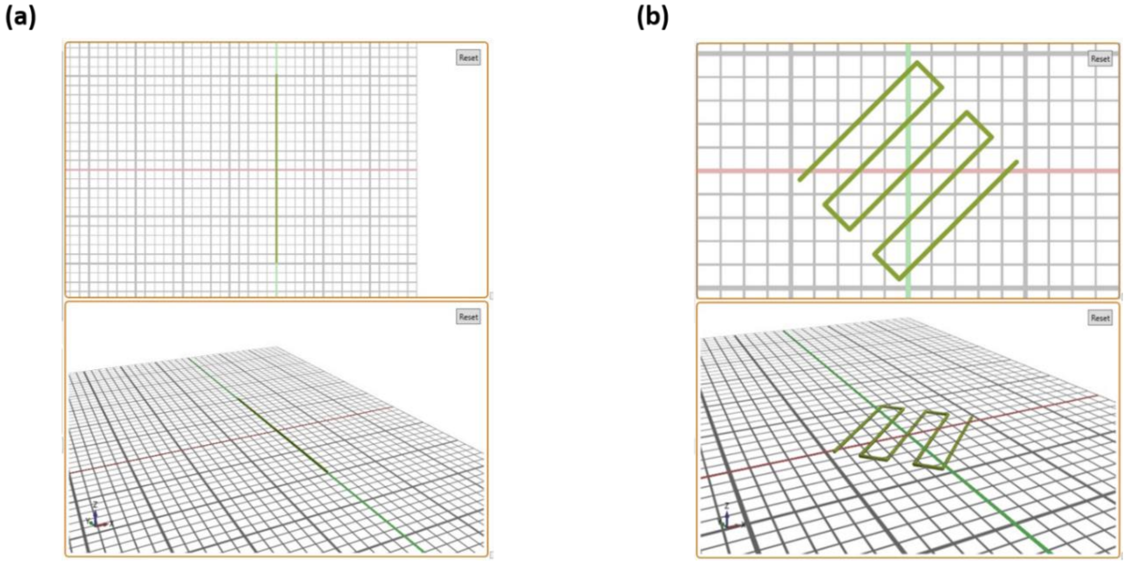

Figure 1. Design of 3D printed coaxial structures including (a) strands, (b) patches.

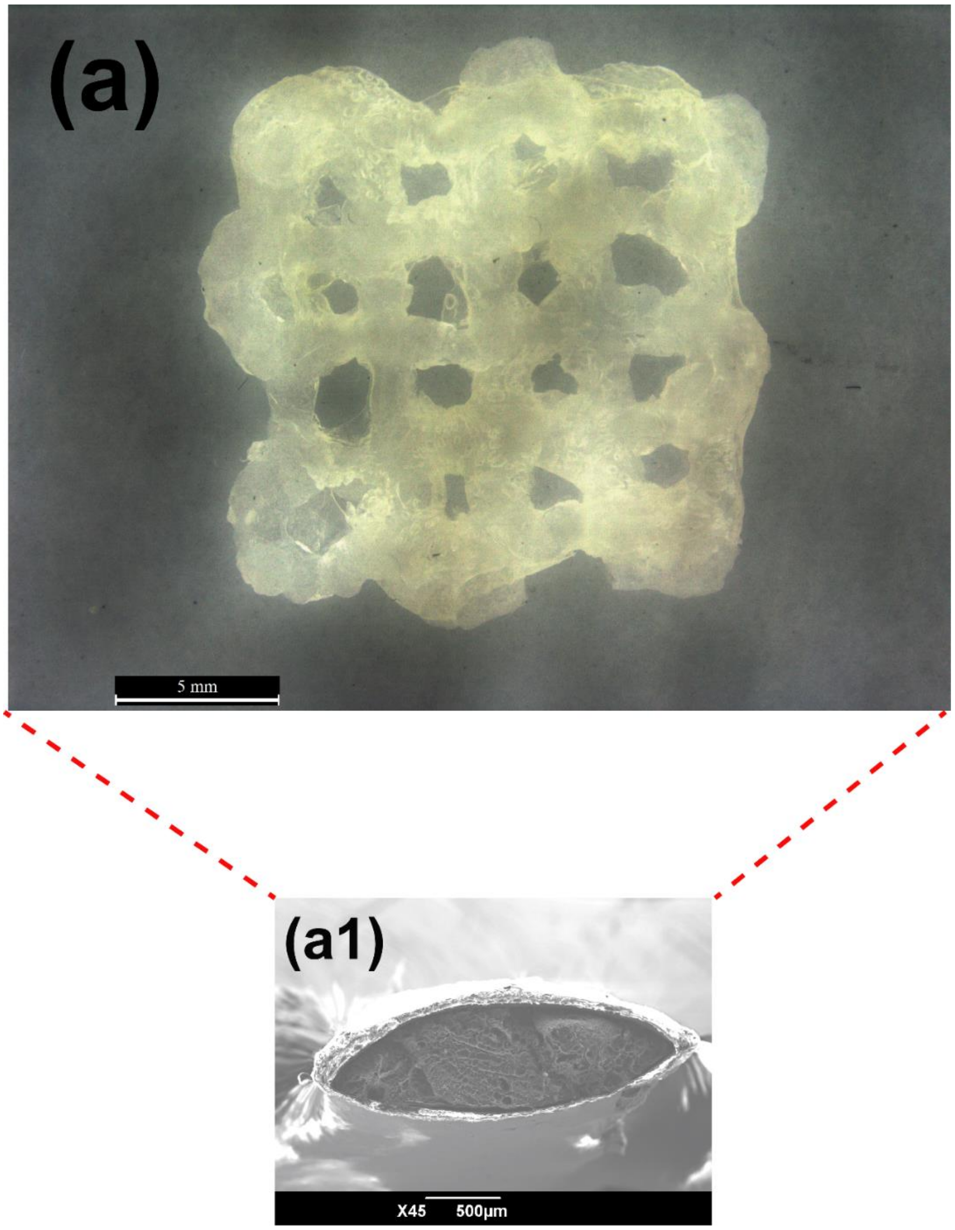

Figure S2. Light microscopy (a) and (a1) SEM images of PLA coated 3D printed coaxial patches containing $CaCO_3$.

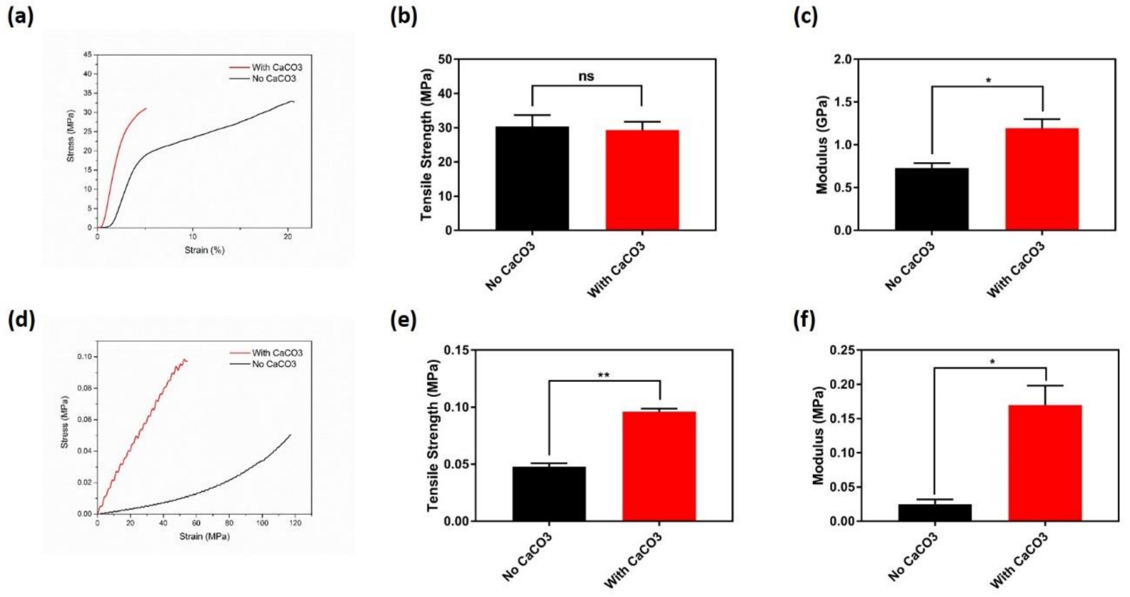

Figure S3. Mechanical properties of 3D printed coaxial strands following a static tensile test, (a-c) Stress-strain curve, tensile strength, and modulus of strands in dry state (n = 3, mean ± SD). (d-f) Stress-strain curve, tensile strength, and modulus of strands in wet state (n = 3, mean ± SD). (*P = 0.05, **P ≤ 0.01).

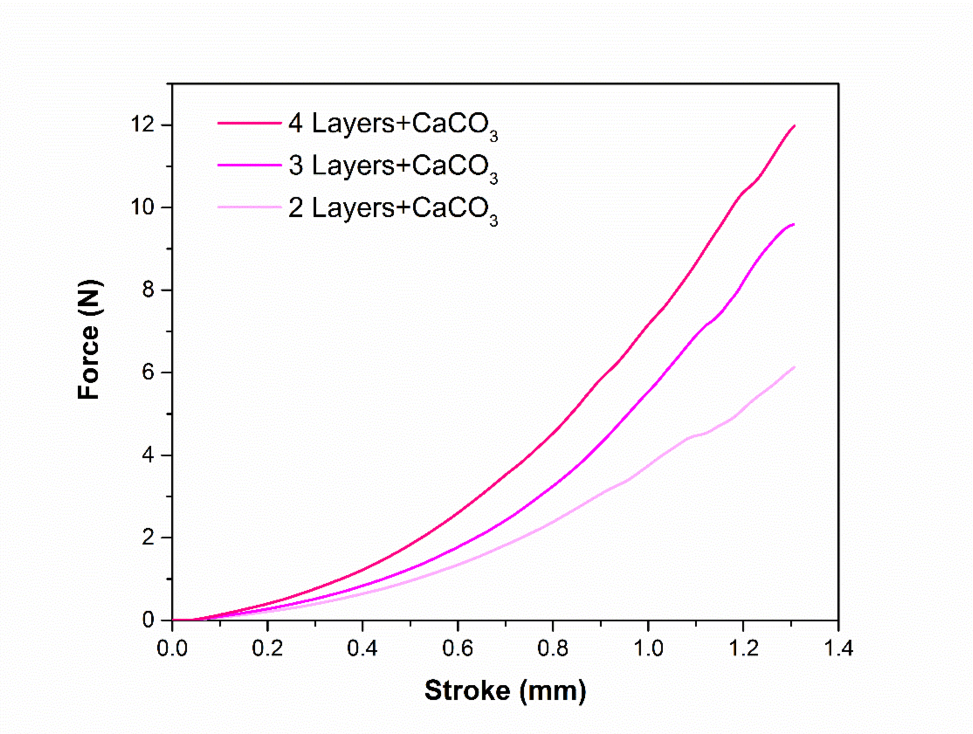

Figure S4. Static compression testing of 3D printed coaxial patches containing $CaCO_3$.

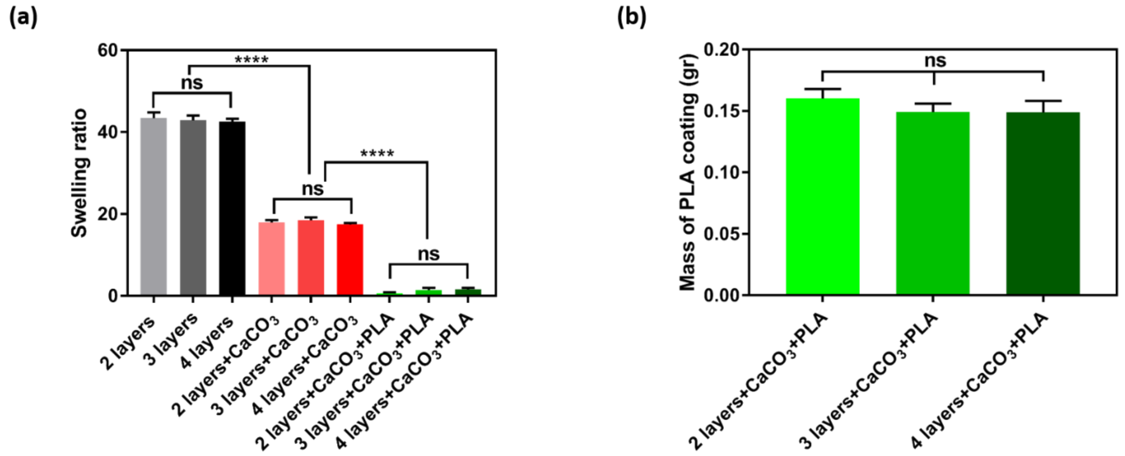

Figure S5. (a) Swelling ratio of different 3D printed coaxial patches in simulated body fluid (SBF). (b) Mass of PLA coating on the 3D printed coaxial patches containing $CaCO_3$, showing no significant difference between 2-, 3-, or 4-layers structures.

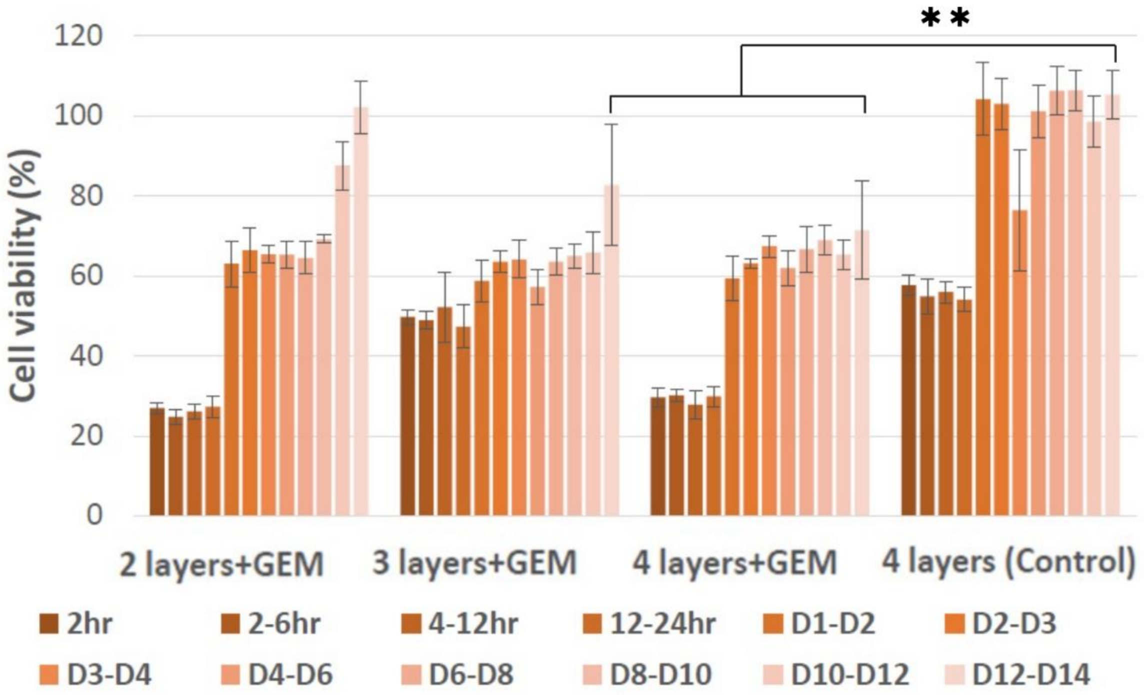

Figure S6. Therapeutic effect of PLA coated 3D printed coaxial patches containing $CaCO_3$, with Gemcitabine (+ GEM) or without Gemcitabine (control), on inhibition of MIA-PaCa-2 pancreatic cancer cell growth. Values are the mean (±SEM) of quadruplicate. D in the figure legend denotes days of treatment. $^{**}P \leq 0.01$.
